# Metformin Therapy and Risk of Cancer in Patients with Type 2 Diabetes: Systematic Review

**DOI:** 10.1371/journal.pone.0071583

**Published:** 2013-08-02

**Authors:** Monica Franciosi, Giuseppe Lucisano, Emanuela Lapice, Giovanni F. M. Strippoli, Fabio Pellegrini, Antonio Nicolucci

**Affiliations:** 1 Department of Clinical Pharmacology and Epidemiology, Consorzio Mario Negri Sud, S. Maria Imbaro (CH), Chieti, Italy; 2 Department of Clinical and Experimental Medicine, University of Naples Federico II, Naples, Italy; 3 Diaverum Medical Scientific Office, Lund, Sweden; 4 School of Public Health, University of Sydney, Darlington, Australia; 5 University of Bari, Bari, Italy; University of Tor Vergata, Italy

## Abstract

**Aims/Hypothesis:**

Diabetes treatments were related with either an increased or reduced risk of cancer. There is ongoing debate about a potential protective action of metformin. To summarize evidence on the association between metformin and risk of cancer and cancer mortality in patients with diabetes.

**Methods:**

Data source: MEDLINE and EMBASE (January 1966-April 2012). We selected randomized studies comparing metformin and other hypoglycaemic agents and observational studies exploring the association between exposure to metformin and cancer. Outcomes were cancer mortality, all malignancies and site-specific cancers.

**Results:**

Of 25307 citations identified, 12 randomized controlled trials (21,595 patients) and 41 observational studies (1,029,389 patients) met the inclusion criteria. In observational studies there was a significant association of exposure to metformin with the risk of cancer death [6 studies, 24,410 patients, OR:0.65, 95%CI: 0.53-0.80], all malignancies [18 studies, 561,836 patients, OR:0.73, 95%CI: 0.61-0.88], liver [8 studies, 312,742 patients, OR:0.34; 95%CI: 0.19-0.60] colorectal [12 studies, 871,365 patients, OR:0.83, 95%CI: 0.74–0.92], pancreas [9 studies, 847,248 patients, OR:0.56, 95%CI: 0.36–0.86], stomach [2 studies, 100701 patients, OR:0.83, 95%CI: 0.76–0.91], and esophagus cancer [2 studies, 100694 patients, OR:0.90, 95%CI: 0.83–0.98]. No significant difference of risk was observed in randomized trials. Metformin was not associated with the risk of: breast cancer, lung cancer, ovarian cancer, uterus cancer, prostate cancer, bladder cancer, kidney cancer, and melanoma.

**Conclusions/Interpretation:**

Results suggest that Metformin might be associated with a significant reduction in the risk of cancer and cancer-related mortality. Randomized trials specifically designed to evaluate the efficacy of metformin as an anticancer agent are warranted.

## Introduction

Type 2 Diabetes Mellitus (DM2) and cancer share many risk factors and there is evidence that type 2 diabetes affects the risk of developing a variety of cancers [1-5]. Several cohort studies show increased cancer incidence and mortality in people diabetes. In particular, an association between DM2 and the risk of colorectal, pancreatic, and breast cancer has been consistently described. An increased risk of liver, gastric, and endometrial malignancies has also been suggested [1].

The increased cancer risk is likely to be related to the interplay between obesity, diabetes and cancer, with hyperinsulinemia playing a crucial role [6]. Insulin can influence tumorigenesis directly, acting on the insulin/insulin like growth factors receptors [7], or indirectly influencing other modulators such as sex hormones, insulin-like growth factors and adipokines [8,9].

Glucose lowering drugs can also variably affect circulating insulin levels. In particular, metformin, the drug of choice for the management of DM2 [10], reduces levels of both circulating glucose and insulin in patients with insulin resistance and hyperinsulinemia. The primary mode of action is through reduced hepatic glucose output [11] via an LKB1/ AMP-activated protein kinase–mediated mechanism. Metformin-induced initiation of an LKB1-mediated AMP-activated protein kinase–dependent energy stress response has been shown to adversely affect the survival of cancer cell lines [12,13]. Metformin also improves insulin sensitivity in peripheral tissue [14,15] reducing hyperinsulinemia.

The anticarcinogenic effects of metformin have been attributed to several mechanisms: activation of LKB1/AMPK pathway, induction of cell cycle arrest and/or apoptosis, inhibition of protein synthesis, inhibition of the unfolded protein response (UPR), activation of the immune system, and a possible eradication of cancer stem cells [16] The activation of LKB1/AMPK pathway inhibits mammalian target of rapamycin (mTOR). This inhibition negatively affects protein synthesis in cancer cells [16]

Evidence from both in vitro and in vivo studies indicates that metformin may inhibit cancer cell growth and reduce cancer risk of some solid tumours. Results from epidemiological studies point out that metformin can reduce the risk of breast, colon, pancreas, and liver cancers, and might improve cancer prognosis, although these data have never been formally summarized [17].

We systematically review available evidence on the association between exposure to metformin and the risk of various forms of cancer and cancer mortality in people with DM2.

## Methods

We conducted the systematic review according to the Preferred Reporting Items for Systematic Reviews and Meta-Analyses (PRISMA) Statement and Cochrane Collaboration guidelines [18] (Table S1).

### Data Sources and Searches

We searched Medline and Embase (January 1966 to April 2012) for randomized and observational studies of the association between metformin and cancer in patients with diabetes mellitus.

We limited searches to studies in humans published in English-language journals. The complete search strategy is reported as Appendix S1.

Reference lists of identified studies, reviews, meta-analyses and other relevant publications were scrutinized to find additional pertinent studies.

### Study Selection

We selected the following study designs: a) prospective, randomized, controlled, open or blinded trials (RCTs) enrolling patients with diabetes mellitus allocated to metformin treatment or a control group (active control or placebo); b) cohort studies, case control or nested case control studies of patients with diabetes mellitus that reported data on exposure to metformin therapy and cancer incidence/prevalence or cancer mortality; c) studies in which exposure to metformin was assessed from prescription databases, and incidence of cancer was derived from cancer registries.

Where more than one publication of one study existed, we used the most complete dataset/recent publication.

We excluded studies with a treatment/exposure duration of less than 24 weeks, or studies in which patients were treated with metformin for other conditions, such as polycystic ovarian syndrome or metabolic syndrome.

### Data Extraction and Quality Assessment

Two authors (MF, EL) independently reviewed results of the search strategy and identified eligible studies; studies that were not published as full reports, such as letters to editor, conference proceedings and commentaries were excluded.

For all eligible studies two authors (MF, EL) independently extracted data using predefined data extraction forms. Information was collected on study design, mean age of study population and percentage of men, intervention/exposure, methodological quality of the studies, number of events and total number of participants in each group.

For observational studies, we selected the results from unadjusted and fully adjusted models (adjusted for the largest number of potential confounders), and recorded the number of cases and total number at risk (for cohort studies) or controls (for case–control studies).

Data on the following dichotomous endpoints were extracted: any cancer death, risk of all malignancies and site-specific cancers including breast, liver, colon, pancreas, stomach, oesophagus, ovary, prostate, lung, kidney, melanoma, uterus, and bladder.

Discrepancies in data extraction between the two reviewers were resolved by discussion and consensus with an arbitrator (AN).

Methodological quality of RCTs was assessed with the risk of bias tool, exploring the following domains: random sequence generation, allocation concealment; blinding of investigators, participants, and outcome assessors; use of intention to treat analysis; completeness of follow-up [18]. For observational studies, we explored selection of study participants, prognostic factor and outcome measurement, adjustment for confounding, and the quality of analysis [19].

### Data Synthesis and Analysis

Risks with 95% confidence intervals were defined according to a hierarchy based on the available risk measures (namely Odds Ratio, Hazards Ratio, Relative Risk, Incidence Rate Ratio). According to the study design, adjusted risks were used for observational studies, while crude risks were used for RCTs.

We pooled risk estimates from individual studies by using random effects models [20]. We used the heterogeneity χ^2^ (Cochran Q) statistic and the I^2^ test to formally analyze heterogeneity across included studies [21]. Presence of publication bias for observational studies was assessed by Begg’s method (Kendall’s Tau) [22]. All analyses were reported separately according study design (RCTs vs. observational studies).

Subgroup analyses and random effects univariate meta-regression were performed where possible to explore the role of the following potential sources of treatment effect heterogeneity, which were defined a priori: age, gender, type of comparison, and follow-up [<1 year vs. ≥ 1year]. As for the type of comparison, based on information reported in individual studies, metformin was compared with no metformin (metformin on top of other treatments vs. same treatments without metformin, or metformin vs. no treatment), with other specific classes of drugs (i.e. metformin vs. sulpholnylureas/tiazolidinediones/insulin), or metformin vs. other unspecified drugs.

Number of events and total patients per arm where reported when available from the original articles.

Pooled risks were reported as Odds Ratios (ORs) and 95% confidence intervals. All analyses were carried out using a macro routine written in SAS language (SAS Release 9.2, Cary, NC, USA. 2002-2008).

This project received no specific external funding. The authors had full responsibility for data collection, data interpretation, and reporting.

## Results

**Figure 1:**
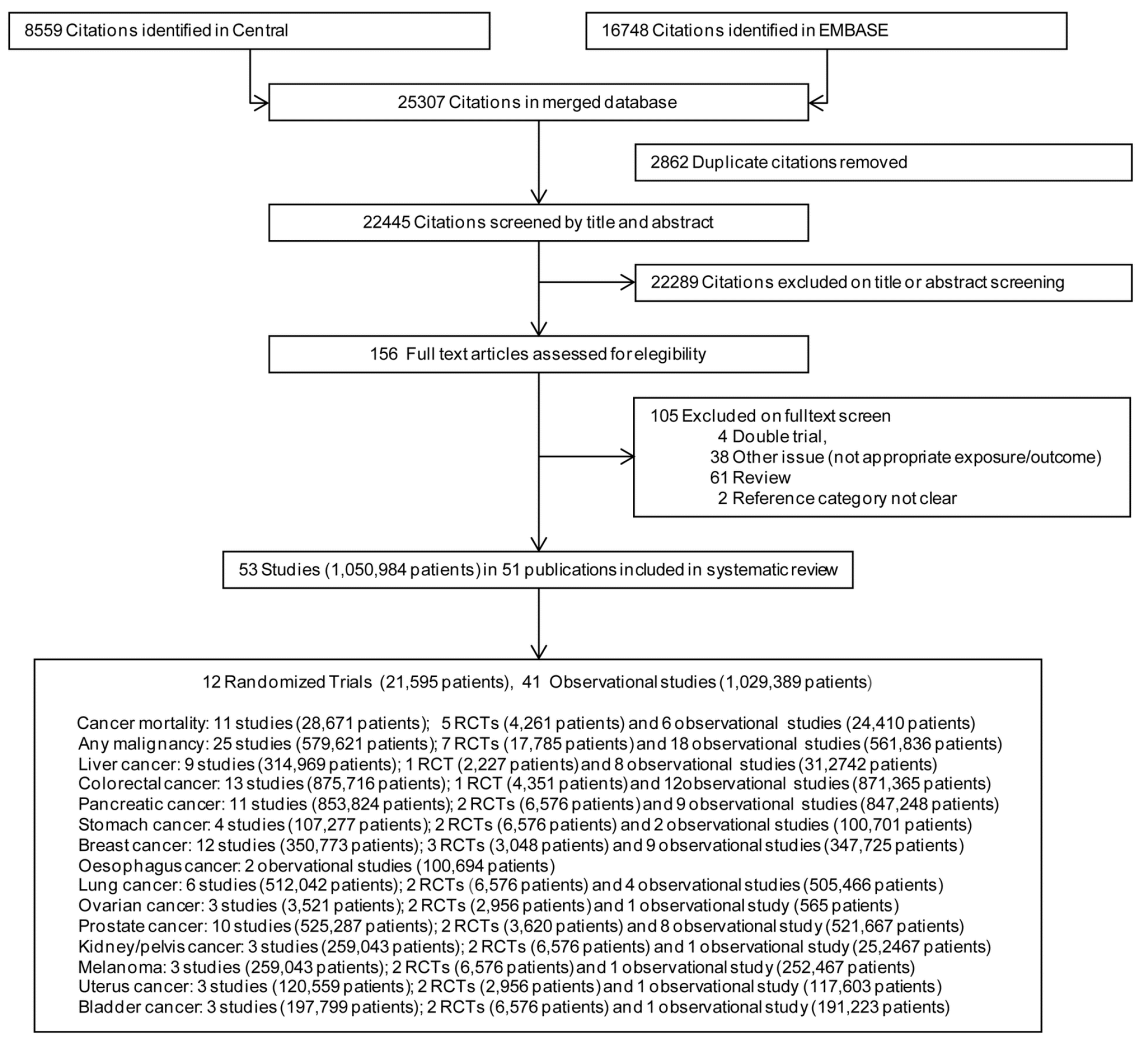
Flow-chart of the study.

We identified a total of 25,307 citations, of which 22,289 were excluded at initial title and abstract screening and 116 after detailed review of the full paper (Figure 1).

We included in the meta-analysis results of 51 eligible articles [23-73] reporting the results of 53 studies (1,029,389 patients). We included 12 RCTs (10 RCTs plus observational extension of 2RCT), 12 case-control studies, 8 nested case-control studies, 21 longitudinal studies (11 retrospective and 10 prospective) (Table S2).

### Risk of bias

In observational studies, according to contemporary standards, study participation (32 studies), prognostic factor (21 studies), outcome measurement (32 studies), and methods for analysis (34 studies) were at low risk of bias, while adjustment for confounding was incomplete or unclear in 33 studies (Figure S1).

Trials were overall of high quality (Figure S2) with the method of sequence generation, details of allocation concealment and blinding at low risk of bias (8, 10, 12 studies, respectively) (Figure S2). There was low risk of bias as a result of outcome reporting and selective outcome reporting in all 12 trials.

### Outcomes

Characteristics of the populations and interventions in studies included in the meta-analysis are reported in Table S2.

### Cancer mortality

**Figure 2:**
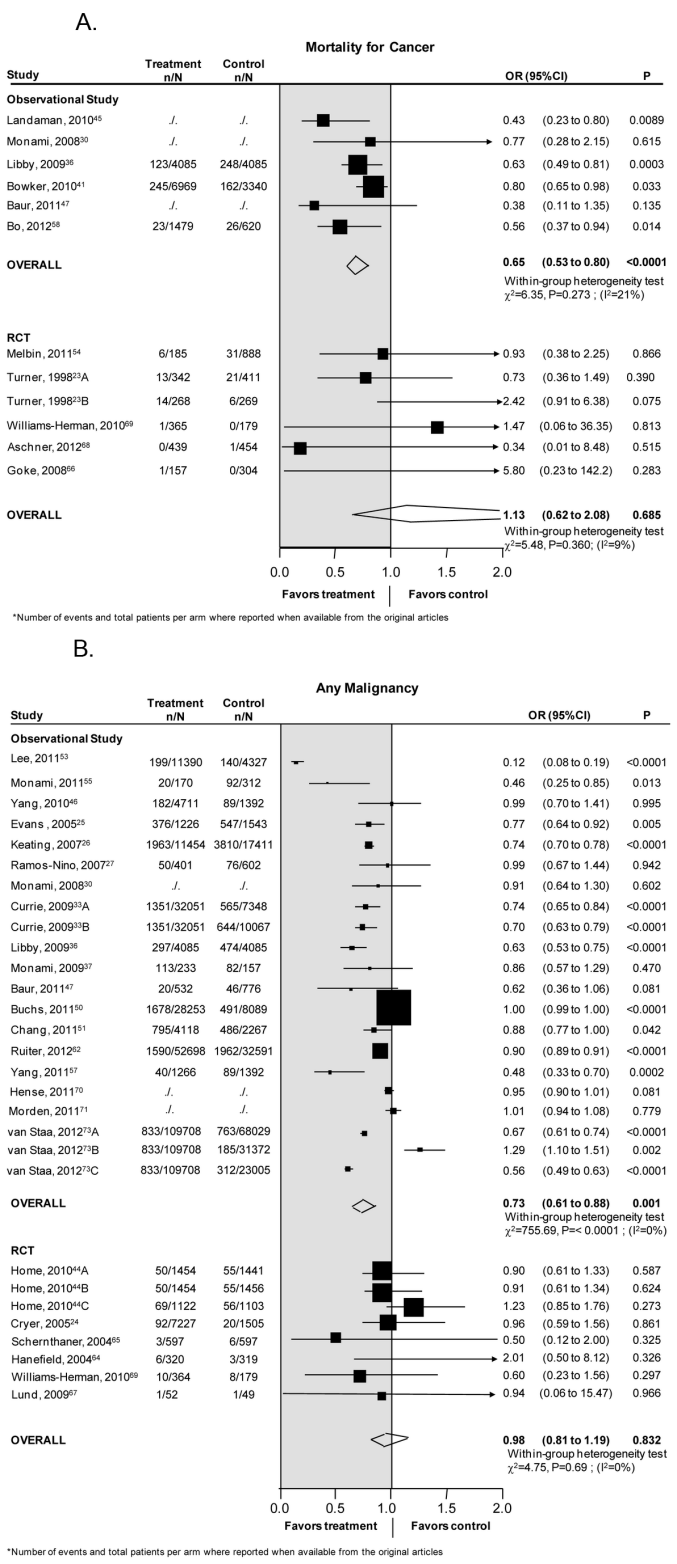
(A) Mortality for Cancer. (B) All Malignancy.

The association between the use of metformin and cancer related mortality was reported in 11 studies recruiting 28,671 patients, 5 RCTs (4,261 patients) [23,54,66,68,69], and 6 observational studies (24,410 patients) [30,36,41,45,47,58] (Figure 2A). Observational studies found a significant reduction in the risk of death due to cancer in patients exposed to metformin compared to patients not exposed to metformin (OR 0.65, 95% CI 0.53 to 0.80, p<0.0001). There was moderate heterogeneity in this analysis (heterogeneity χ^2^=6.35, p=0.27, I^2^=21%). Heterogeneity could be explained primarily by age (100% of explained heterogeneity, p=0.035). Analyses stratified by comparisons showed that the reduction in the risk of cancer-related mortality was present when metformin was compared to no metformin [OR 0.59, CI 0.35-1.0, p=0.05, heterogeneity χ^2^=4.52, p=0.10, I^2^=56%], and when metformin was compared to no use of glucose lowering drugs [OR 0.59, CI 0.39–0.90, p=0.02, heterogeneity χ^2^=0.31, p=0.56, I^2^=0%].

There was no significant difference in the risk of death due to cancer with metformin compared to no metformin in RCTs.

Begg’s method suggests the absence of publication bias (p=0.13).

### Any malignancy

The association between the use of metformin and the risk of any malignancy was described in 25 studies recruiting 579,621 patients, 7 RCTs (17,785 patients) [24,44,64,65,67,69], and 18 observational studies (561,836 patients) [25-27,30,33,36,37,46,47,50,51,53,55,57,62,70,71,73] (Figure 2B).

Existing observational studies found a significant reduction in the risk of any malignancy in patients exposed to metformin compared to patients not exposed to metformin [OR 0.73, CI 0.61-0.88, p=0.001]. However, there was a high degree of heterogeneity among the studies (heterogeneity χ^2^=755.7, p<0.0001, I^2^=97%).

There was no significant difference in the risk of any malignancy with metformin compared to no metformin in RCTs (0.98; 0.81-1.19, p=0.83).

Only a small amount of the variance was explained by the characteristics tested in the meta-regression (age explained 1.9% of the variance, p=0.0005). Similar risk reductions were detected when metformin therapy was compared with no metformin (OR 0.82, CI 0.72-0.94; heterogeneity χ^2^=129.59, p<0.0001, I^2^=92) or with sulphonylureas (OR 0.77, CI 0.65-0.92; heterogeneity χ^2^=41.4, p<0.0001, I^2^=95). The risk reduction associated with metformin therapy was also evident when compared to other hypoglycemic agents, although statistical significance was not reached [OR 0.41; CI 0.12-1.39, p=0.15, heterogeneity χ^2^=57.7 p<0.0001, I^2^=97%].

Begg’s method suggests the absence of publication bias (p=0.65).

### Liver cancer

**Figure 3:**
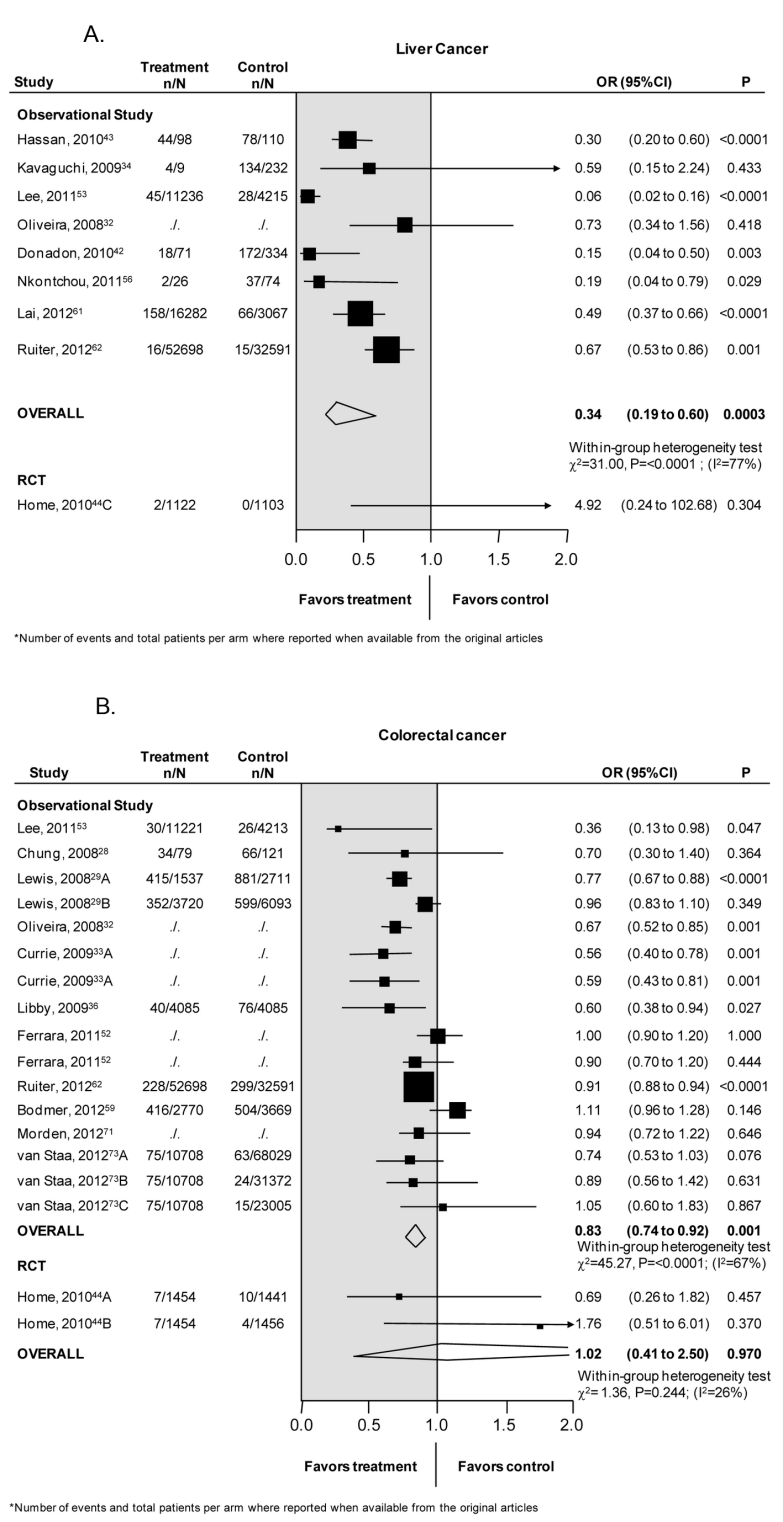
(A) Liver Cancer. (B) Colorectal cancer.

Nine studies, 1 RCT (2,227 patients) [44], and 8 observational (312,742 patients) [32,34,42,43,53,56,61,62] examined the association between metformin and liver cancer (Figure 3A). Observational studies found a significant reduction in the risk of liver cancer associated with the use of metformin [OR 0.34; CI 0.19-0.60, p=0.0003]. There was a high degree of heterogeneity among the studies (heterogeneity χ^2^=31.0, p=0.0001, I^2^=77%).

Subgroup meta-analysis according to the different comparisons confirmed the overall estimates for metformin vs. no metformin [OR 0.44, CI 0.32-0.62, p<0.001, heterogeneity χ^2^=5.39, p=0.25, I^2^=26], while the risk of liver cancer was decreased by 91% when metformin was compared to other drugs [OR 0.09, CI 0.04-0.21, p<0.001, heterogeneity χ^2^=1.21, p=0.27, I^2^=17%]. The comparison of metformin vs. sulphonylureas showed a 44% risk reduction [OR 0.56, CI 0.34-0.91, p=0.02] (heterogeneity χ^2^=2.47, p=0.12, I^2^=59%).

Begg’s methods suggested the absence of publication bias (p=0.089).

### Colorectal cancer

Thirteen studies assessed the association between metformin and colorectal cancer: 1 RCT (4,351 patients) [44], and 12 observational: (871,365 patients) [28,29,32,33,36,52,53,59,62,71,73]. Observational studies showed that the risk of colorectal cancer was decreased by 17% [OR 0.83, CI 0.74–0.92, p=0.0009] among patients treated with metformin, as compared to those not using metformin (Figure 3B). There was a moderate degree of heterogeneity among the studies analyzed (heterogeneity χ^2^=45.3, p<0.0001, I^2^=67%). No association was found between risk of colorectal cancer and use of metformin in RCTs. [OR 1.02, CI 0.41-2.5, p=0.97]

Heterogeneity was not explained by the predefined characteristics tested in univariate metaregression.

Subgroup meta-analysis confirmed the protective effect associated with metformin therapy when compared to other hypoglycemic agents [OR 0.55, CI 0.36-0.83, p=0.005, heterogeneity χ^2^=0.82, p=0.36, I^2^=0%] or to sulphonylureas [OR 0.75, CI 0.56-1.0, p=0.05, heterogeneity χ^2^=9.6, p=0.008, I^2^=79%]. The risk reductions associated with metformin therapy was also evident when compared to no metformin [OR 0.90; CI 0.80-1.02, p=0.09, heterogeneity χ^2^=21.93, p=0.003, I^2^=68%], or to insulin [OR 0.75, CI 0.43-1.31, p=0.0.31, heterogeneity χ^2^=3.1, p=0.08, I^2^=67%], although statistical significance was not reached

Begg’s method suggested the presence of publication bias in observational studies (p= 0.089).

### Pancreatic cancer

**Figure 4:**
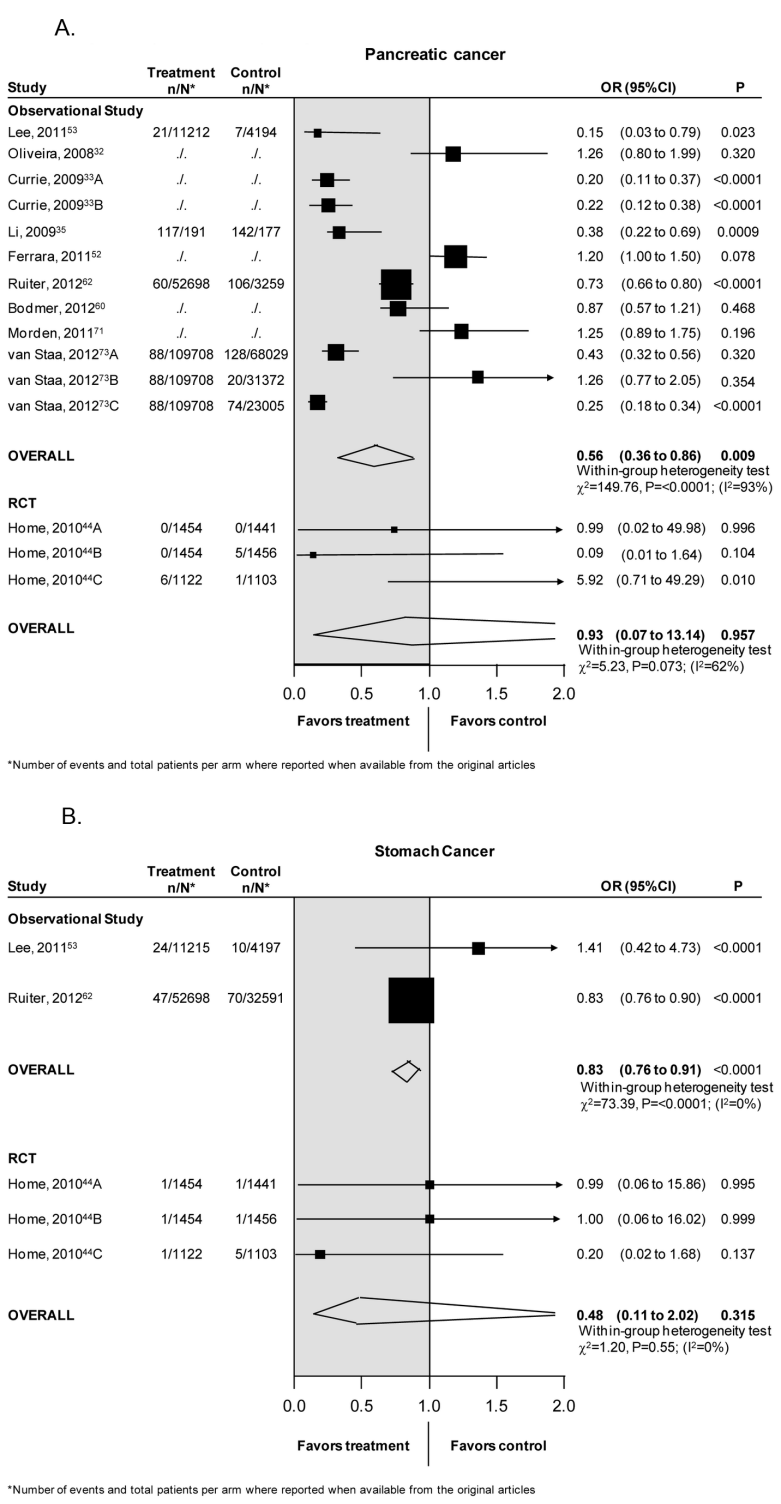
(A) Pancreatic cancer. (B) Stomach Cancer.

Eleven studies assessed the association between metformin and pancreatic cancer: 2 RCTs (6,575 patients) [44], and 9 observational studies (847,248 patients) [32,33,35,52,53,60,62,71,73]. Observational studies showed that the use of metformin was associated with a 44% reduction in the risk of pancreatic cancer [OR 0.56, CI 0.36–0.86, p=0.009], with a high degree of heterogeneity among the studies (heterogeneity χ^2^=149.8, p<0.0001, I^2^=93%) (Figure 4A).

The risk reduction associated with the use of metformin was not significant in RCTs (OR 0.93, CI 0.07-13.14, p=0.95).

Heterogeneity was not explained by the predefined characteristics tested in univariate metaregression.

Stratification according to the type of comparisons confirmed the protective role of metformin when compared to sulphonylureas [OR 0.42, CI 0.21-0.84, p=0.02, heterogeneity χ^2^=29.5, p<0.0001, I^2^=93%], or to insulin [OR 0.24, CI 0.18-0.32, p<0.0001, heterogeneity χ^2^=0.19, p=0.67, I^2^=0%]. No significant reduction was detected when metformin was compared to no metformin [OR 0.95; CI 0.64-1.40, p=0.79, heterogeneity χ^2^=16.29, p=0.003, I^2^=75%].

Begg’s method suggested the absence of publication bias (p=0.22).

### Stomach cancer

Four studies, 2 RCTs (6,576 patients) [44] and 2 observational (100,701 patients) [53,62] reported the association of metformin therapy with stomach cancer (Figure 4B). The meta-analysis of observational studies showed that the use of metformin was associated with a significant reduction in the risk of stomach cancer [OR 0.83, CI 0.76–0.91, p<0.0001], without heterogeneity across studies (heterogeneity χ^2^=0.73, p=0.39, I^2^=0%) (Figure 4B). No significant reduction was detected when pooling the results of RCTs (0.48, 0.11-2.02, p=0.31).

### Oesophagus cancer

Two observational studies reported the association between metformin therapy and oesophagus cancer in 100,694 patients. The random effects meta-analysis showed that the use of metformin was associated with a significant reduction in the risk of cancer of the oesophagus [OR 0.90 CI 0.83–0.98, p=0.013] without heterogeneity across the studies (heterogeneity χ^2^=0.60, p=0.44, I^2^=0%) (Figure S3).

### Breast cancer

Twelve studies, 3 RCTs (3,048 patients) [44,63] and 9 observational (347,725 patients) [33,36,40,49,52,62,71-73] reported the risk of breast cancer associated with metformin therapy.

Observational studies showed that the use of metformin was associated with a non significant reduction in the risk of breast cancer [OR 0.97, CI 0.88-1.08, p=0.58] (Figure S4). No significant effect emerged from RCTs (1.49, 0.74-2.98, p=0.27).

Subgroup meta-analyses showed a significant reduction in the risk of breast cancer only when metformin was compared with other drugs [OR 0.71, CI 0.58-0.88, p=0.001] without heterogeneity across studies (heterogeneity χ^2^=0.99, p=0.61, I^2^=0%].

### Prostate cancer

Ten studies evaluated the association of metformin therapy on prostate cancer: 2 RCTs (3,620 patients) [44] and 8 observational studies (521,667 patients) [31,33,38,39,52,62,71,73].

Both observational studies and RCTs showed that the use of metformin had no effect on the risk of prostate cancer (Figure S5).

### Lung cancer

Six studies, 2 RCTs (6,576 patients) [44], and 4 observational studies (505,466 patients) [36,51,62,73] reported the association of metformin therapy and the risk of lung cancer. Observational studies showed that the use of metformin was associated with a marginally non significant reduction in the risk of lung cancer [OR 0.83, CI 0.64–1.06, p=0.13], (heterogeneity χ^2^=17.91, p=0.003, I^2^=72%) (Table S3). No significant reduction in the risk of lung cancer was documented in RCTs (0.73, 0.37-1.45, p=0.38).

### Ovarian cancer

Three studies, two RCTs (2,956 patients) [44] and one observational (565 patients) [47], examined the association between use of metformin and ovarian cancer. No statistically significant association was found in both RCTs and observational studies (Table S3).

### Other forms of cancer

Three studies recruiting 259,043 patients, 2RCTs [44] and 1 observational [52] reported the association of metformin therapy and kidney/pelvis cancer, melanoma, or uterus, and three studies involving 197,799 patients, 2 RCTs [44] and 1 observational [32] reported the association of metformin therapy and bladder cancer. No significant effect of metformin on the risk of these cancers was found (Table S3).

## Discussion

### Keys findings

We show in a comprehensive systematic review that in observational studies metformin use might be associated with a significant reduction in the risk of several forms of cancers, including colorectal, liver, pancreatic, stomach, and oesophagus cancer. We found no significant association between metformin use and risk of other neoplasms such as prostate, breast, kidney, melanoma, uterus, ovarian, lung, and bladder cancer.

In relative terms, exposure to metformin was associated with a 35% reduction in the risk of cancer mortality, and a 31% reduction in the risk of any cancer.

Such potential anti-tumor effect of metformin, identified in observational studies, is yet unproven in the few existing randomized trials of metformin as an intervention. These trials have been never primarily designed to evaluate the effect of metformin on such outcomes, and therefore confirmation is required in ‘ad hoc’ designed intervention studies.

### Comparison with other studies

Our results are coherent with previous observations suggesting a protective role of metformin for cancer and expand the results of previous meta-analyses. There have been 7 meta-analyses of existing studies of the association of metformin with different types of cancer in patients with diabetes [74-80]. Comparison between existing analyses and ours is difficult for methodological reasons. We considered more outcomes, identified a much larger number of studies and patients, and analyzed separately observational studies and RCTs. Furthermore, we adopted more rigorous criteria for study selection; in fact, some of the previous meta-analyses also included studies with individuals without diabetes as the reference category [75,77,79]. Furthermore, previous analyses did not provide details of literature search strategies and did not provide explicit assessment of risk of bias in the identified studies.

### Strengths and weaknesses

The major strength of our systematic review is represented by the up to date, large number of studies and patients included, together with the full spectrum of cancers considered. We separately analyze the results of various observational study designs and randomized trials, providing separate estimates of data and identifying areas of unmet needs, particularly in the lack of randomized trials which tested metformin as an anti-cancer agent. We also perform an analysis of risk of bias for observational studies based upon current validated methods. We document that the protective effect of metformin is not uniform across the different neoplasms considered, being particularly strong for liver and pancreatic cancers, relevant for colorectal and stomach cancers. Of note, metformin seems to be particularly effective in preventing some of the neoplasms associated with a particularly poor prognosis, and this translates into a significant 35% reduction in cancer related mortality.

One limitation of our findings is they are primarily based on results derived from observational studies, which are unpredictably prone to bias and confounding inherent to their design.

In particular, some of the studies included were retrospective, and interviewer and recall bias could lead to an overestimate of effect [81]. In addition, some of the studies were based on medical data or insurance data that were not specifically designed to assess the effect of metformin therapy on cancer. Details on dose, duration, variation over time for treatments as well as full information on risk factors and potential confounders were incomplete. The possibility of immortal bias was not completely ruled out in some included studies. In addition, the presence of indication bias cannot be excluded. It is possible that metformin users had a shorter duration of diabetes (baseline lower risk of cancer), even if most of these studies reported analysis adjusted for these confounding, minimizing this potential bias. Furthermore, the presence of publication bias cannot be ruled out for colorectal and pancreatic cancers. We also explored comprehensively the availability of randomized trials on the topic, but found only few, with imprecise estimates, due to the fact that these were not primarily designed to explore the proposed research and clinical question.

Another limitation of our analysis is the heterogeneity of the comparator populations. In particular, in some studies the comparator group was defined as “no metformin users”, including patients treated with any other glucose lowering medications. Among these classes of drugs, the most common were insulin and sulphonylureas, both associated with hyperinsulinemia and probably with an increased risk of cancer. Thus, the effect of metformin therapy could be overestimated as compared to the potential hazardous effect associated with other agents in the reference group.

The diversity in study populations, comparators, and study design translated into a substantial heterogeneity in effect estimates across studies for many of the analyses performed. Nevertheless, in all cases heterogeneity was quantitative, rather than qualitative in nature. In fact, almost all the studies showed a protective effect of metformin, though variable in magnitude.

### Future research

Recent evidence from retrospective data, suggests a more favourable outcome among patients with T2DM and breast or lung cancer treated with metformin [82,83]. It is plausible that the predominant mechanism of metformin action will differ across patient characteristics and types of cancers. Thus, metformin can exert its action involving a broad range of different therapeutic target and markers. A better understanding of the mechanisms involved can help identify the patients who might benefit from metformin.

Currently, a number of clinical trials examining the use of metformin as a cancer therapy are underway, including studies in prostate, breast, colorectal, endometrial and pancreatic cancer. These trials, together with new pathophysiological studies, will contribute in elucidating the role of metformin as an anticancer agent.

## Supporting Information

Appendix S1 Search strategy. (DOC)

Figure S1 Risk of bias in randomized clinical trials. (PDF)

Figure S2 Risk of bias in observational studies. (PDF)

Figure S3 Oesophagus Cancer. (PDF)

Figure S4 Breast Cancer. (PDF)

Figure S5 Prostate Cancer. (PDF)

Table S1 PRISMA checklist. (DOC)

Table S2 Characteristics of the study included in the Meta-analyses. (DOC)

Table S3 Results of additional meta-analysis. (DOC)
