# Supplementary material for: Metformin Therapy and Risk of Cancer in Patients with Type 2 Diabetes: Systematic Review
Source: PLoS One. 2013 Aug 2;8(8):e71583. doi: 10.1371/journal.pone.0071583 (PMC3732236; doi:10.1371/journal.pone.0071583)
Supplement: Appendix S1 — (DOC) [file pone.0071583.s001.doc]

| [#1](http://www.ncbi.nlm.nih.gov/pubmed/advanced)4 | Search **#13 NOT Animal** | [8559](http://www.ncbi.nlm.nih.gov/pubmed/?cmd=HistorySearch&querykey=13) |
| --- | --- | --- |
| [#13](http://www.ncbi.nlm.nih.gov/pubmed/advanced) | Search **#9 AND (#7 OR #8)** Filters: **Publication date from 1966/01/01 to 2012/04/30; Humans; English** | [8763](http://www.ncbi.nlm.nih.gov/pubmed/?cmd=HistorySearch&querykey=13) |
| [#12](http://www.ncbi.nlm.nih.gov/pubmed/advanced) | Search **#9 AND (#7 OR #8)** Filters: **Publication date from 1966/01/01 to 2012/04/30; Humans** | [9797](http://www.ncbi.nlm.nih.gov/pubmed/?cmd=HistorySearch&querykey=12) |
| [#11](http://www.ncbi.nlm.nih.gov/pubmed/advanced) | Search **#9 AND (#7 OR #8)** Filters: **Publication date from 1966/01/01 to 2012/04/30** | [10031](http://www.ncbi.nlm.nih.gov/pubmed/?cmd=HistorySearch&querykey=11) |
| [#10](http://www.ncbi.nlm.nih.gov/pubmed/advanced) | Search **#9 AND (#7 OR #8)** | [10093](http://www.ncbi.nlm.nih.gov/pubmed/?cmd=HistorySearch&querykey=10) |
| [#9](http://www.ncbi.nlm.nih.gov/pubmed/advanced) | Search **#4 AND #5** | [29838](http://www.ncbi.nlm.nih.gov/pubmed/?cmd=HistorySearch&querykey=9) |
| [#8](http://www.ncbi.nlm.nih.gov/pubmed/advanced) | Search **"Randomized Controlled Trials as Topic"[Mesh] OR "Randomized Controlled Trial" [Publication Type] OR randomized[ti/ab] OR randomised[ti/ab]) OR random[ti/ab] OR “double-blind”[ti/ab] OR placebo[ti/ab]** | [433216](http://www.ncbi.nlm.nih.gov/pubmed/?cmd=HistorySearch&querykey=8) |
| [#7](http://www.ncbi.nlm.nih.gov/pubmed/advanced) | Search **("epidemiologic studies"[mesh] OR "epidemiologic studies"[All Fields] OR "epidemiological studies"[All Fields] OR “cohort studies”[mesh] OR ("cohort"[text word] AND “study”[text word]) “cohort studies”[All Fields] OR "risk"[mesh] OR "case-control studies"[mesh] OR ("case-control"[text word] AND “study”[text word]) OR "case-control study"[All Fields] OR ("case"[text word] AND "control"[text word] AND “study”[text word]) OR "case control study"[All Fields] OR “longitudinal studies”[MESH] OR “longitudinal studies”[All Fields] OR ("longitudinal"[text word] AND “study”[text word]) OR “retrospective studies”[MESH] OR “retrospective studies”[All Fields] OR ("retrospective"[text word] AND “study”[text word]) OR "prospective"[text word] OR "observational"[All Fields] OR "cohort"[All Fields] OR (“case”[All Fields] AND “control”[All Fields]) OR "case control"[text word] OR “cross-sectional studies”[mesh] OR “cross sectional”[All Fields] OR “non-randomized”[All Fields] OR “nonrandomized”[All Fields] OR “non-randomised”[All Fields] OR “nonrandomised”[All Fields]** | [2244808](http://www.ncbi.nlm.nih.gov/pubmed/?cmd=HistorySearch&querykey=7) |
| [#5](http://www.ncbi.nlm.nih.gov/pubmed/advanced) | Search **("diabetes mellitus"[mesh] OR diabetes[ti/ab] OR diabetic[ti/ab])** | [303699](http://www.ncbi.nlm.nih.gov/pubmed/?cmd=HistorySearch&querykey=5) |
| [#4](http://www.ncbi.nlm.nih.gov/pubmed/advanced) | Search **("Hypoglycemic Agents"[Majr] OR "Biguanides"[Majr] OR "Metformin"[Majr] OR Hypoglycemic[Text Word] OR Hypoglycaemic[Text Word] OR Biguanides[Text Word] OR Biguanide[Text Word] OR Metformin[Text Word])** | [55103](http://www.ncbi.nlm.nih.gov/pubmed/?cmd=HistorySearch&querykey=4) |

**Appendix 1: Search strategy**

MEDLINE

**#6**

**#5** NOT **Animal**

EMBASE

16,748

**#5**

**#3** AND **#4**

[16,936](http://embase.com.library.marionegri.it/search/results?viewsearch=4)

**#4**

**#1** AND **#2**

[68,525](http://embase.com.library.marionegri.it/search/results?viewsearch=4)

**#3**

**'case control study'**/exp OR **'case control'** OR **'prospective study'**/exp OR **prospective** OR **'observational study'**/exp OR **observational** OR **'retrospective study'**/exp OR **retrospective** OR **'randomized controlled trial'**/exp OR **randomized** OR **randomised** OR **random** OR **'placebo'**/exp OR **'double-blind'** AND [humans]/lim AND [english]/lim AND [embase]/lim AND [1-1-1966]/sd NOT [30-4-2012]/sd

[985,442](http://embase.com.library.marionegri.it/search/results?viewsearch=3)

**#2**

**'diabetes mellitus'**/exp OR **'diabetes'**/exp OR **'diabetic'**/exp AND [humans]/lim AND [english]/lim AND [embase]/lim AND [1-1-1966]/sd NOT [30-4-2012]/sd

[243,653](http://embase.com.library.marionegri.it/search/results?viewsearch=2)

**#1**

**'antidiabetic agent'**/exp/mj OR **'antidiabetic'**/exp OR **'hypoglycemic'**/exp OR **hypoglycaemic** OR **'metformin'**/exp/mj OR **'metformin'**/exp OR **'biguanide derivative'**/exp/mj OR **'biguanide'**/exp OR **'biguanides'**/exp AND [humans]/lim AND [english]/lim AND [embase]/lim AND [1-1-1966]/sd NOT [30-4-2012]/sd

[127,788](http://embase.com.library.marionegri.it/search/results?viewsearch=1)
