# Supplementary figures and images for: Metformin Therapy and Risk of Cancer in Patients with Type 2 Diabetes: Systematic Review
Source: PLoS One. 2013 Aug 2;8(8):e71583. doi: 10.1371/journal.pone.0071583 (PMC3732236; doi:10.1371/journal.pone.0071583)

**Figure S1: Risk of bias in randomized clinical trials**

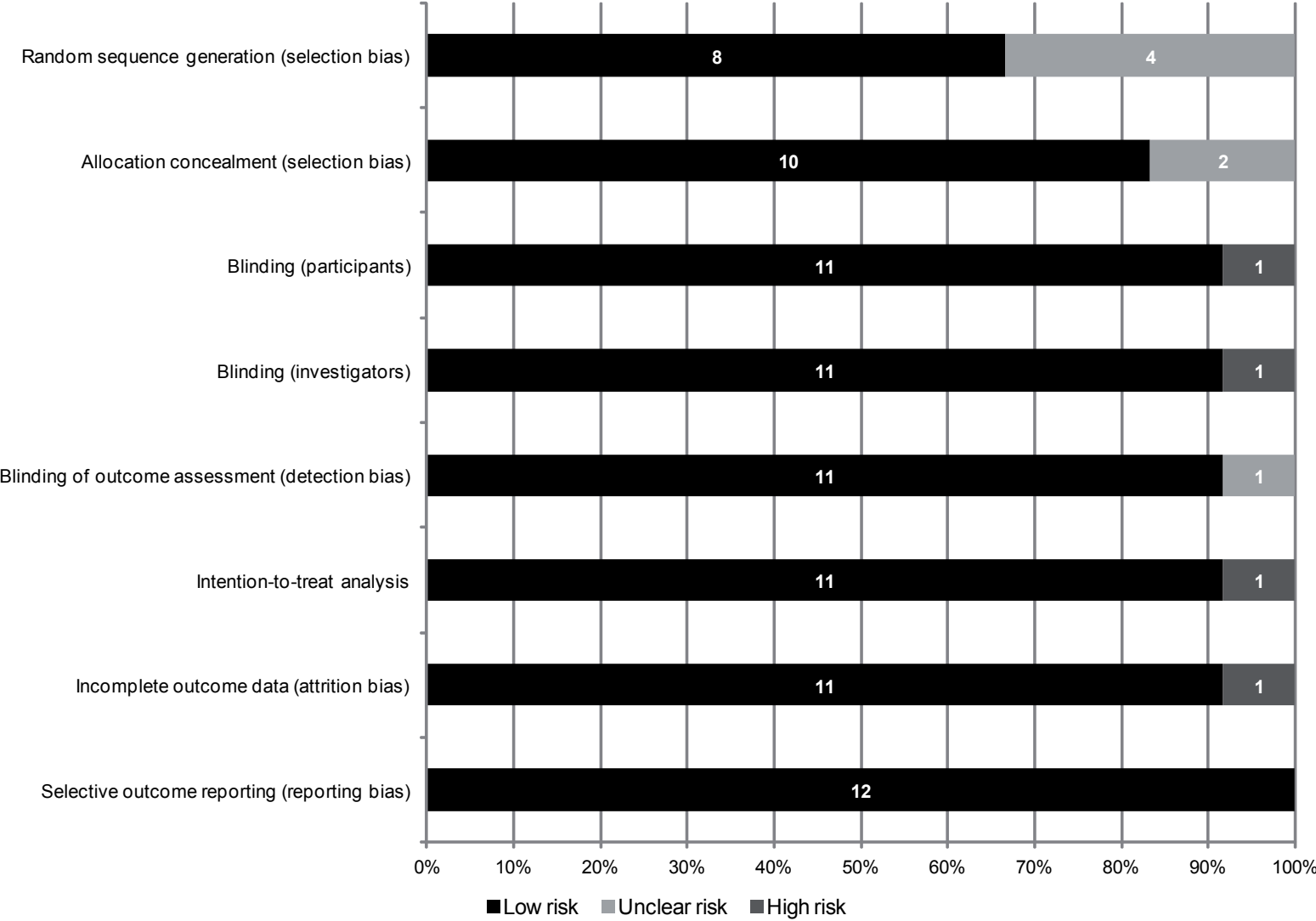

Supplement: Figure S1 — (PDF) [file pone.0071583.s002.pdf]

Figure S2: Risk of bias in observational studies

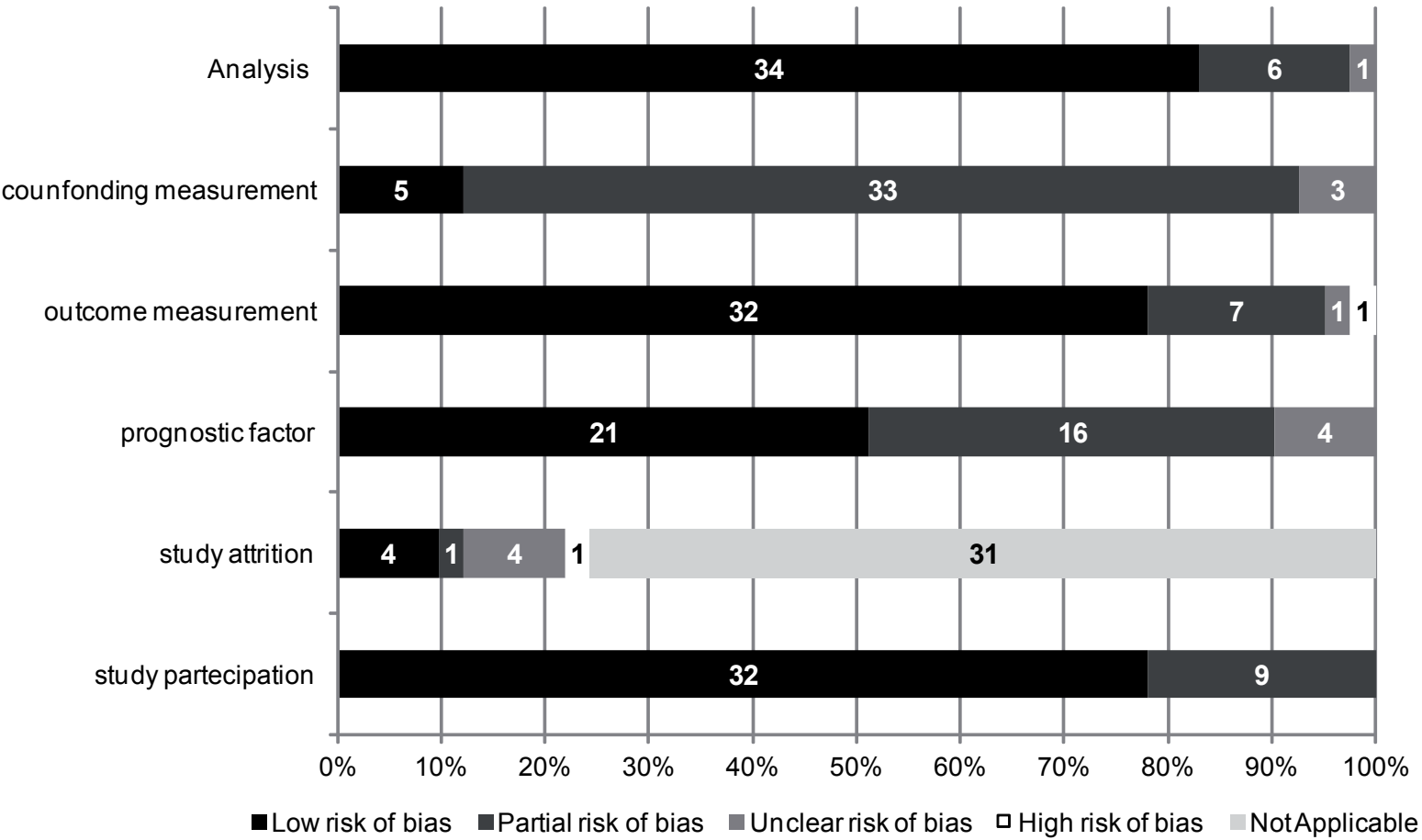

Supplement: Figure S2 — (PDF) [file pone.0071583.s003.pdf]
