# Supplementary material for: Metformin Therapy and Risk of Cancer in Patients with Type 2 Diabetes: Systematic Review
Source: PLoS One. 2013 Aug 2;8(8):e71583. doi: 10.1371/journal.pone.0071583 (PMC3732236; doi:10.1371/journal.pone.0071583)
Supplement: Figure S3 — (PDF) [file pone.0071583.s004.pdf]

Figure S3: Oesophagus cancer: results of Meta-analyses based on 2 observational studies (100694 patients)

Oesophagus Cancer

Study	Treatment n/N*	Control n/N*	OR (95%CI)	P
**Observational Study**				
Lee, 2011[53]	21/11212	6/4193	0.44 (0.07 to 2.61)	0.374
Ruiter, 2012[62]	45/52698	46/32591	0.90 (0.82 to 0.97)	0.314
**OVERALL**			**0.90 (0.83 to 0.98)**	**0.013**

Heterogeneity test
χ^2=0.60, P=0.439; (I^2=0%)

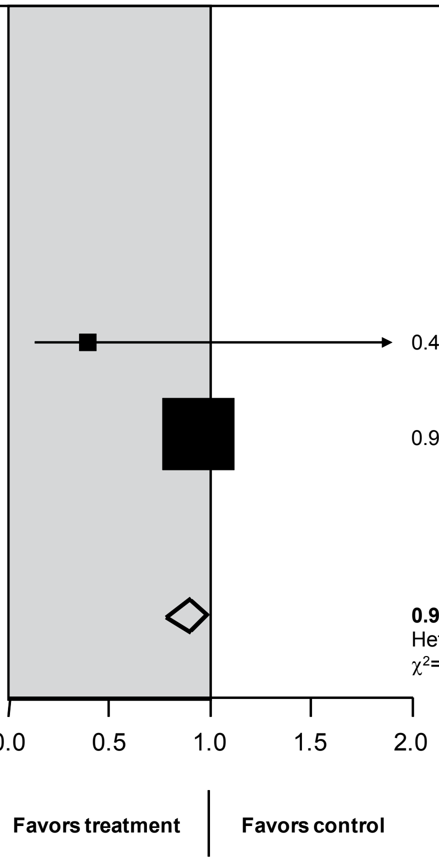

*Number of events and total patients per arm where reported when available from the original articles
