# Supplementary material for: Metformin Therapy and Risk of Cancer in Patients with Type 2 Diabetes: Systematic Review
Source: PLoS One. 2013 Aug 2;8(8):e71583. doi: 10.1371/journal.pone.0071583 (PMC3732236; doi:10.1371/journal.pone.0071583)
Supplement: Figure S4 — (PDF) [file pone.0071583.s005.pdf]

Figure S4: Breast cancer: results of Meta-analyses based on 9 observational studies (347225 patients) and 3 RCTs (3048 patients)

Breast Cancer

Study	Treatment n/N*	Control n/N*	OR (95%CI)	P
**Observational Study**				
Bosco, 2011[49]	96/1250	297/3073	0.81 (0.63 to 0.96)	0.058
Currie, 2009[33]A	./.	./.	1.02 (0.71 to 1.45)	0.058
Currie, 2009[33]B	./.	./.	0.93 (0.69 to 1.26)	0.529
Libby, 2009[36]	24/1848	41/1875	0.60 (0.32 to 1.10)	0.823
Bodmer, 2010[40]A	165/778	140/680	1.03 (0.76 to 1.39)	0.240
Bodmer, 2010[40]B	62/260	64/314	1.22 (0.82 to 1.82)	0.668
Ferrara, 2011[52]	./.	./.	0.90 (0.80 to 1.00)	0.990
Ruiter, 2012[62]	207/28266	217/16892	0.95 (0.91 to 0.98)	0.990
Morden, 2011[71]	./.	./.	1.28 (1.05 to 1.57)	0.016
Chlebowski, 2012[72]	./.	./.	0.65 (0.45 to 0.93)	0.018
van Staa 2012[73]A	86/47913	49/29837	1.09 (0.77 to 1.55)	0.619
van Staa 2012[73]B	86/47913	18/13398	1.33 (0.80 to 2.22)	0.263
van Staa 2012[73]C	86/47913	15/10163	1.22 (0.70 to 2.11)	0.484
**OVERALL**			**0.97 (0.88 to 1.08)**	**0.584**
			Within-group heterogeneity test χ^2=23.12, P=0.027; (I^2=1%)	
**RCT**				
Home, 2010[44]A	6/590	3/605	2.06 (0.51 to 8.28)	0.874
Home, 2010[44]B	6/590	6/645	1.09 (0.35 to 3.41)	0.887
Home, 2010[44]C	8/554	4/562	2.04 (0.61 to 6.83)	0.236
Gregorio, 1999[63]	0/45	1/47	0.34 (0.01 to 8.58)	0.513
**OVERALL**			**1.49 (0.75 to 2.99)**	**0.258**
			Within-group heterogeneity test χ^2=1.56, P=0.647; (I^2=0%)	

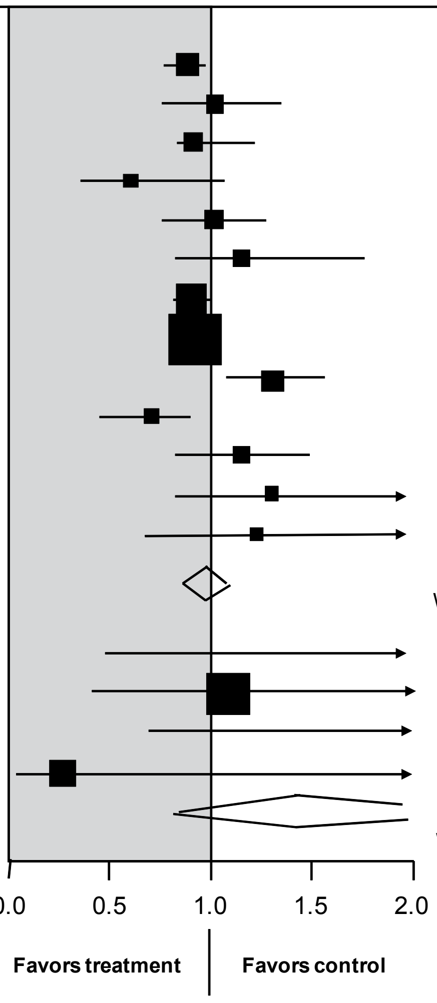

*Number of events and total patients per arm where reported when available from the original articles
