# Supplementary material for: Metformin Therapy and Risk of Cancer in Patients with Type 2 Diabetes: Systematic Review
Source: PLoS One. 2013 Aug 2;8(8):e71583. doi: 10.1371/journal.pone.0071583 (PMC3732236; doi:10.1371/journal.pone.0071583)
Supplement: Figure S5 — (PDF) [file pone.0071583.s006.pdf]

Figure S5: Prostate cancer: results of Meta-analyses based on 8 observational studies (521667 patients) and 2 RCTs (3620 patients)

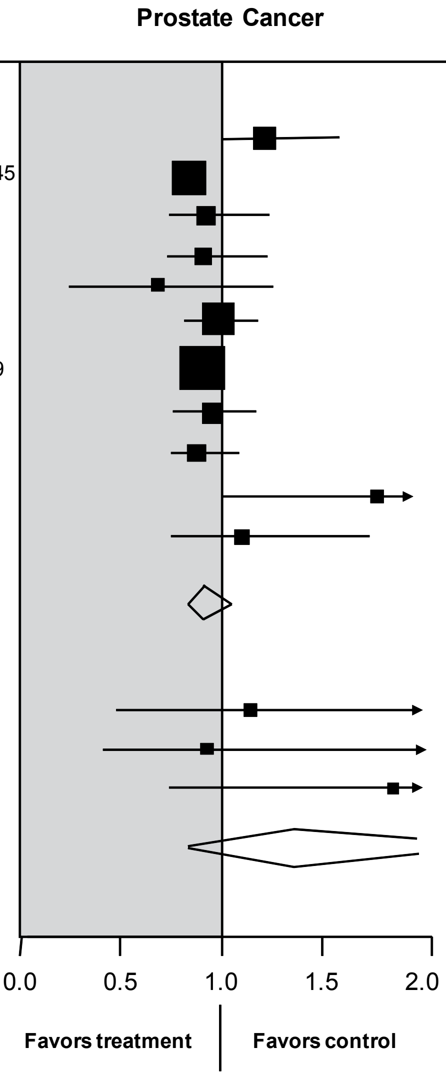

Study	Treatment n/N*	Control n/N*	OR (95%CI)	P
**Observational Study**				
Azoulay, 2010[39]	536/5772	203/2326	1.23 (0.99 to 1.52)	0.058
Murtola, 2008[31]	964/2101	23759/47345	0.80 (0.73 to 0.88)	<0.0001
Currie, 2009[33]A	./.	./.	0.93 (0.67 to 1.32)	0.694
Currie, 2009[33]B	./.	./.	0.91 (0.66 to 1.27)	0.568
Wright, 2009[38]	40/85	57/113	0.69 (0.35 to 1.38)	0.289
Ferrara, 2011[52]A	./.	./.	1.00 (0.90 to 1.10)	1.000
Ruiter, 2012[62]	90/24432	136/15699	0.92 (0.88 to 0.97)	0.001
Morden, 2011[71]	./.	./.	0.97 (0.76 to 1.24)	0.807
van Staa, 2012[73]A	98/61795	70/38192	0.87 (0.64 to 1.18)	0.355
van Staa, 2012[73]B	98/61795	17/17974	1.68 (1.00 to 2.81)	0.049
van Staa, 2012[73]C	98/61795	19/13842	1.07 (0.66 to 1.75)	0.782
**OVERALL**			**0.96 (0.87 to 1.05)** Within-group heterogeneity test χ^2=24.91, P=0.006; (I^2=60%)	**0.365**
**RCT**				
Home, 2010[44]A	10/864	9/836	1.08 (0.44 to 2.66)	0.874
Home, 2010[44]B	10/864	10/811	0.94 (0.39 to 2.27)	0.887
Home, 2010[44]C	10/568	5/541	1.92 (0.65 to 5.66)	0.236
**OVERALL**			**1.18 (0.69 to 2.04)** Within-group heterogeneity test χ^2=1.08, P=0.582; (I^2=0%)	**0.545**

*Number of events and total patients per arm where reported when available from the original articles
