# Supplementary material for: Metformin Therapy and Risk of Cancer in Patients with Type 2 Diabetes: Systematic Review
Source: PLoS One. 2013 Aug 2;8(8):e71583. doi: 10.1371/journal.pone.0071583 (PMC3732236; doi:10.1371/journal.pone.0071583)
Supplement: Table S2 — (DOC) [file pone.0071583.s008.doc]

| **Author year**  **Table S1: Characteristics of the study included in the Meta-analyses** | **Study desingn** | **Country** | **Source population:**  **setting, register or hospital** | **Age** | **Males** | **Outcome** | **Treatment comparison** | **Site of cancer (n°events/n°patients)** | **Measure of outcome** | **Adjustment** |
| --- | --- | --- | --- | --- | --- | --- | --- | --- | --- | --- |
| Turner, 1998^23^A | UKPDS: multi-centre randomized trial | UK | Patients with diabetes enrolled in 15 centers. Median Follow-up 10.7 yrs | 53 | 46.5 | ICD9 codes (140-239) | Non hypoglicaemic drurg users | Cancer mortality (34/753) | OR | No adjustment variables |
| Turner, 1998^23^B | UKPDS: multi-centre randomized trial | UK | Patients with diabetes enrolled in 15 centers. Median Follow-up 10.7 yrs | 59 | 60 | ICD9 codes (140-239) | Sulfonylureas users | Cancer mortality (20/537) | OR | No adjustment variables |
| Cryer, 2005^24^ | COSMIC: multicentre, randomized , open-label controlled clinical trial | USA | Patients with diabetes randomized to metformin or usual care. One year of Follow-up | 58.4 | 49.3 | Reported as Serious Advers Events | Usual care (diet or sulfonylureas) | All malignancy (112/8732) | OR | No adjustment variables |
| Evans , 2005^25^ | Case-control study | UK | T2DM patients in diabetes clinical information system (DARTS) and a database of dispensed prescriptions (MEMO) record linkage databases from 1993-to 2003. | 73 | 53 | ICD-9 or ICD-10  (Not listed) | Non metformin users | All malignancies (923/2769) | AOR | Matching for age, year of diagnosi, sex.  AOR adjusted for smoking, BMI, Blood pressure, postcode rak for material deprivation |
| Keating, 2007^26^ | Case-control study | USA | Patients with diabetes in Kaiser Permanente Of Northern California database from 1994 to 2001 | 65.3 | 52.9 | Kaiser Permanente Cancer registery | Non metformin users | All malignancies (5773/28865) | OR | No adjustment variables |
| Ramos-Nino, 2007^27^ | Case-control study | USA | Patients with diabetes in charge of primary care in Vermont Diabetes Information system from 2003 to 2005 | 64.8 | 45.6 | (Patients’ )Self reporting of any cancer, leukemia lymphoma | Non metformin users | All malignancies (126/1003) | OR | No adjustment variables |
| Chung, 2008^28^ | Case-control study | Korea | Patients with T2DM underwent to total colonoscopy in Gastrointestinal endoscopy unit- Hallym University Hospital- from 2003-to 2006 | 66.5 | 52 | Colonoscopic examination | Non metformin users | Colon (100/200) | AOR | Age, sex, BMI, duration of diabetes, HbA1c, lipids, insulin and aspirin therapy |
| Lewis, 2008^29^A | Nested case-control study | USA | Patients with diabetes in Kaiser Permanente of Northern California from 1999 to 2005 | 71 | 53 | Colonoscopy ≥1 adenomatous polyps or invasive colorectal cancer | Non metformin users | Colon (1296/4248) | OR | No adjustment variables |
| Lewis, 2008^29^B | Nested case-control study | USA | Patients with diabetes in Kaiser Permanente of Northern California from 1999 to 2005 | 66.1 | 54.9 | Sigmoidoscopy | Non metformin users | Colon (951/9813) | OR | No adjustment variables |
| Monami, 2008^30^ | Retrospective cohort study | Italy | Patients with T2DM referring to University diabetes clinic-Florence from 2000 to 2005. Median of Follow-up 4.3 yrs | 63.4 | 53.9 | ICD codes (140-210 and 229-239) | Other hypoglicaemic drug users | Cancer mortality NA/1171) | AOR | Age, sex, BMI Charles comorbidity |
|  |  |  |  | 63.4 | 53.9 | ICD codes (140-210 and 229-239 | All malignancies (NA/1171) | All malignancies (NA/1171) | AOR |  |
| Murtola, 2008^31^ | Case-control study | Finland | Patients with diabetes in Finnish Cancer registry from 1995-2002 | 68 | 100 | Hystologically confirmed | Non metformin users | Prostate cancer (2101/47345) | AOR | Age, place of residency, and simultaneous use of other medication (aspirin, cholesterol-lowering drugs, or anthihypertensive drugs) |
| Oliveira, 2008^32^ | Retrospective cohort study. | USA | Patients with diabetes with pharmacy and medical benefit coverage in geographically diverse areas from 2000 to 2004 | 56 | 51 | Algorithm based on ICD9 codes plus procedures and treatment | Non metformin users | Colon (383/191223) | ARR | History of polyps, ulcerative colitis, Crohn’s disease; |
|  |  |  |  |  |  |  |  | Bladder (178/191223) |  | Schistosomiasis, pelvic radiation; |
|  |  |  |  |  |  |  |  | Liver (39/191223) |  | Hepatitis B/hepatitis C, cirrhosis, alcoholism; |
|  |  |  |  |  |  |  |  | Pancreas (102/191223) |  | Partial gastrectomy, chronic pancreatitis, deep venous thrombosis, dermatomyositis /polymyositis, alcoholism, hepatitis B/hepatitis C,history of polyps |
| Currie, 2009^33^A | Retrospective cohort study | UK | Patients with diabetes in charge of General practices participating in The Health Information Network (THIN)  Follow-up 2.4yrs | 60.78 | 51.8 | THIN database | Sulfonylureas users | All malignancy (196137483) | AHR | Age, gender, smoking, previous cancer |
|  |  |  |  |  |  |  |  | Prostate |  |  |
|  |  |  |  |  |  |  |  | Breast |  |  |
|  |  |  |  |  |  |  |  | Colon |  |  |
|  |  |  |  |  |  |  |  | Pancreas |  |  |
| Currie, 2009^33^B | Retrospective cohort study | UK | Patients with diabetes in charge of General practices participating in The Health Information network (THIN)  Follow-up 2.4yrs | 59.84 | 52.1 | THIN database | Insulin users | All malignancy (1955/40123) | AHR | Age, gender, smoking, previous cancer |
|  |  |  |  |  |  |  |  | Prostate |  |  |
|  |  |  |  |  |  |  |  | Breast |  |  |
|  |  |  |  |  |  |  |  | Colon |  |  |
|  |  |  |  |  |  |  |  | Pancreas |  |  |
| Kavaguchi, 2009^34^ | Nested case-control study | Japan | Patients with diabetes in Hospital based multi-center cohort from 2004-2008 | 67.05 | 67.6 | Ultrasonic guided biopsy or supermagnetic iron oxide-enhanced magnetic resonance imaging | Non metformin users | Liver(138/241) | OR | No adjustment variables |
| Li, 2009^35^ | Case-control study | USA | Patients with diabetes attending MD Anderson Cancer Center from 2004-2008. | 63.7 | 65.7 | Newly diagnosed and pathological confirmed pancreatic adenocarcinoma | Non metformin users | Pancreas(255/361) | AOR | Age, sex , race, smoking, alchohol, BMI, family history of cancer, diabetes duration, and use of insulin |
| Libby, 2009^36^ | Retrospective cohort study | UK | Patients with T2DM in Diabetes Audit and Research in Tayside Scotland (DARTS) from 1994 to 2003 | 66.3 | 54.4 | Scottish morbidity record 6 (SMR6) and Registrar general with ICD9-10 codes | Other hypoglicaemic drug users | All malignancy(771/8170) | AHR | Age, sex, smoking, deprivation,BMI,HbA1c,insulin use, and Sulphonilureas |
|  |  |  |  | 66.3 | 54.4 |  |  | Colon(116/8170) |  |  |
|  |  |  |  | 66.3 | 54.4 |  |  | Lung(93/8170) |  |  |
|  |  |  |  | - | 0 |  |  | Breast(65/3723) |  |  |
|  |  |  |  | 66.3 | 54.4 |  |  | Cancer mortality(371/8170) |  |  |
| Monami, 2009^37^ | Nested case-control study | Italy | Patients with diabetes referring to Diabetes Outpatient Clinic of the Geriatric Unit-University of Florence from 1998 to 2004 | 69.25 | 59 | ICD 9 codes (140-209)  Regional Hospital discharge system and mortality register of Tuscany | Non metformin users | All malignancy (195/390) | OR | No adjustment variables |
| Wright, 2009^38^ | Case-control study | USA | Patients with diabetes in Seattle Peuget Sound SEER cancer registry-from 2002-2005-King county Hospital | 60.79 | 100 | Histologically confirmed Prostate cancer | Non metformin users | Prostate (97/198) | AOR | Age, other diabetic treatment, aspirin, NSAID usage, BMI, PSA test in preceding 5 years and family history of prostate cancer |
| Azoulay, 2010^39^ | Nested Case-Control Study | UK | T2DM patients in the General Practice Research Database (GPRD) from 1988 to 2009 | 74.1 | 100 | Medical code or combination of medical procedure and treatment | Non metformin users | Prostate (739/8098) | ARR | HbA1c, excessive alcohol use, obesity, smoking, lower urinary tract symptoms, previous cancer, and use of NSAIDs, antihypertensive drugs, statins, and other antidiabetic agents |
| Bodmer, 2010^40^A | Nested case-control study | UK | Patients with diabetes in General Practice Research Database (GPRD) from 2004 to 2005 | 67.5 | 0 | Recorded first diagnosis of invasive breast cancer or in situ carcinoma followed b surgery, radiation, chemotherapy antiestrogen therapy or a combination therapy | Non metformin users | Breast (305/1458) | AOR | Age sex, general practice, calendar time by matching, use of prandial glucose regulators, acarbose, thiazolidinediones, estrogens, smoking, BMI, diabetes duration, and A1C |
| Bodmer, 2010^40^B | Nested case-control study | UK | Patients with diabetes in General Practice Research Database (GPRD) from 2004 to 2005 | - | 0 | Recorded first diagnosis of invasive breast cancer or in situ carcinoma followed b surgery, radiation, chemotherapy antiestrogen therapy or a combination therapy | Sulfonylureas users | Breast (126/574) | AOR | Age sex, General practice,calendar time by matching, use of prandial glucose regulators, acarbose, thiazolidinediones, estrogens, smoking, BMI, diabetes duration, and A1C |
| Bowker, 2010^41^ | Retrospective cohort study | Canada | New users of metformin or sulfonylurea in Saskatchewan Health database from 1991 to 1996 ( Administrative databases) | 63.45 | 55.1 | Vital statistic file of Saskatchewan Health | Non metformin users | Cancer mortality (407/10309) | AHR | Age, sex and Chronic disease score (CDS) |
| Donadon, 2010^42^ | Case-control study | Italy | Patients with T2DM attending the 3rd Internal Medicine Pordenone General Hospital from 1994 to 2008 | - | 83.7 | Citological or histological examinations (14.4% ultrasound α-fetroprotein, mtomography scan/magnetic resonance | Non metformin users | Liver (190/405) | AOR | Sex, age, BMI, HBV and HCV infection, alcohol use ALT lever, tryglicerides, cholesterol Diabetes duration,and antidiabetic therapy |
| Hassan, 2010^43^ | Case-control study | USA | Patients with diabetes enrolled in ongoing hospital case control study from 2000 to 2008. University Hospital –Anderson Cancer Center | 63.5 | 74.5 | New diagnosis of HCC treated at the center | Non metformin users | Liver (122/208) | AOR | Age, sex, race, educational level, cigarette smoking, alcohol drinking, hepatitis C virus, hepatitis B virus, family history of cancer |
| Home, 2010^44^A | ADOPT: multicentre double-blind, randomized, controlled clinical trial | USA, Canada and Europe | Type 2 diabetes enrolled in 488 centers in the United States, Canada, and 15 European countries. Follow-up: median of 4.0 yrs. | 57.1 | 58.7 | Repoted as serious advers events | Glibenclamide | All malignancies (105/2895) | OR | No adjustment variables |
|  |  |  |  |  |  |  |  | Breast (9/1195) |  |  |
|  |  |  |  |  |  |  |  | Colorectal (17/2895) |  |  |
|  |  |  |  |  |  |  |  | Stomach (2/2895) |  |  |
|  |  |  |  |  |  |  |  | Pancreatic (0/2895) |  |  |
|  |  |  |  |  |  |  |  | Bladder (8/2895) |  |  |
|  |  |  |  |  |  |  |  | Renal (3/2895) |  |  |
|  |  |  |  |  |  |  |  | Uterus (4/1195) |  |  |
|  |  |  |  |  |  |  |  | Prostate (19/1700) |  |  |
|  |  |  |  |  |  |  |  | Lung (13/2895) |  |  |
|  |  |  |  |  |  |  |  | Melanoma (4/2895) |  |  |
|  |  |  |  |  |  |  |  | Ovary (1/2895) |  |  |
| Home, 2010^44^B | ADOPT: multicentre double-blind, randomized, controlled clinical trial | USA, Canada and Europe | Type 2 diabetes enrolled in 488 centers in the United States, Canada, and 15 European countries. Follow-up: median of 4.0 yrs. | 57.1 | 57.6 | Reported as serious advers events | Rosiglitazone | All malignancy (105/2910) | OR | No adjustment variables |
|  |  |  |  |  |  |  |  | Breast cancer (12/1235) |  |  |
|  |  |  |  |  |  |  |  | Colorectal (11/2910) |  |  |
|  |  |  |  |  |  |  |  | Stomach (2/2910) |  |  |
|  |  |  |  |  |  |  |  | Pancreatic (5/2910) |  |  |
|  |  |  |  |  |  |  |  | Bladder (6/2910) |  |  |
|  |  |  |  |  |  |  |  | Renal (3/2910) |  |  |
|  |  |  |  |  |  |  |  | Uterus (2/1235) |  |  |
|  |  |  |  |  |  |  |  | Prostate (20/1675) |  |  |
|  |  |  |  |  |  |  |  | Lung (11/2910) |  |  |
|  |  |  |  |  |  |  |  | Melanoma (4/2910) |  |  |
|  |  |  |  |  |  |  |  | Ovariy (3/2910) |  |  |
| Home, 2010^44^C | RECORD:  multicentre, randomized ,  open-label controlled clinical trial | Europe, Australia and New Zeland | Type 2 diabetes enrolled in 364 centres in 25 countries in  Europe and Australasia. Mean follow-up duration was 5.5 yrs. | 59.75 | 50.65 | Reported as serious advers events | Rosiglitazone | All malignancy (125/2225) | OR | No adjustment variables |
|  |  |  |  |  |  |  |  | Renal (5/2225) |  |  |
|  |  |  |  |  |  |  |  | Bladder (7/2225) |  |  |
|  |  |  |  |  |  |  |  | Pancreatic (7/2225) |  |  |
|  |  |  |  |  |  |  |  | Stomach (6/2225) |  |  |
|  |  |  |  |  |  |  |  | Liver (2/2225) |  |  |
|  |  |  |  |  |  |  |  | Melanoma (2/2225) |  |  |
|  |  |  |  |  |  |  |  | Lung (12/2225) |  |  |
|  |  |  |  |  |  |  |  | Prostate (15/1109) |  |  |
|  |  |  |  |  |  |  |  | Uterus (14/1116) |  |  |
|  |  |  |  |  |  |  |  | Breast (12/1116) |  |  |
|  |  |  |  |  |  |  |  | Ovary (4/2225) |  |  |
| Landman, 2010^45^ | Retrospective cohort study | Netherlands | Patients with diabetes enrolled in Zwolle outpatient diabetes project integrating available care (ZODIAC)- General practice and hospital based specialist nurse –median of Follow-up 9.6 yrs | 67.8 | 42.4 | Vital status assessmant | Non metformin users | Cancer mortality (122/1353) | AHR | Smoking (yes or no), age, sex, diabetes duration, A1C, serum creatinine, BMI, blood pressure, total cholesterol– to–HDL ratio, albuminuria, insulin use, sulfonylurea use, and macrovascular complications (yes or no). |
| Yang, 2010^46^ | Prospective cohort study | Hong Kong-China | T2DM patients in Hong Kong Diabetes Registery Hong Kong Diabetes Registery from 1996-to 2005. - median of Follow-up 4.9 yrs | 57.4 | 46.01 | ICD9 codes (140-208) | Non metformin users | All malignancies (271/6103) | AHR | Age, sex, body mass index, smoking status, alcohol use status, HbA1c, SBP, LDL-C related risk (i.e., <2.80 mmol/L plus albuminuria and 3.80 mmol/L), spline functions (non-linear associations) of HDL-C and triglyceride for cancer, ever statins usage and ever RAS inhibitor usage, ever metformin usage and ever insulin usage. |
| Baur, 2011^47^ | Prospective cohort study  Detect-study | Germany | Patient with diabetes in DETECT-study (Primary Care Practice) from sept2003 to 2008 | 66.6 | na | ICD-10 (ode not specified) | Non metformin users | All malignancy (66/1308) | A HR | Age, sex, smoking status, BMI at baseline |
|  |  |  |  |  |  | Clinical report confirmed by Death certificates |  | Cancer mortality (32/1308) |  |  |
| Bodmer, 2011^48^ | Case-control study | UK | Patients with diabetes in General Practice Research Database (GPRD) from 1995 to 2009 | - | 0 | READ medical Codes (not listed) plus chemiotheray or radiotherapy or specialized oncology visit | Non metformin users | Ovary (85/555) | AOR | BMI smoking, prior use of estrogen and/or oral contraceptive, hystory of hysterectomy>3 yrs befor index date, endometriosi and/or polycistic ovaries, HbA1c, and diabetes duration |
| Bosco, 2011^49^ | Nested case-control study | Denmark | Patients with diabetes in the Danish National Registry from 1989 to 2009 | 70.5 | 0 | ICD8-ICD10 codes | Non metformin users | Breast (393/4323) | AOR | adjust for confounding and selection bias introduced by matching on county and adjusting for complications due to diabetes, clinical |
| Buchs, 2011^50^ | Prospective cohort study | Israel | Patients with diabetes in Maccabi Healthcare Service (MHS) from 2003 to 2007. Median follow-up 4.5yrs | >65 yrs (39.1%) | 54.95 | International classification of diseases for oncology or specific drugs for cancer | Non metformin users | All malignancy (2169/36342) | AHR | Age, sex, glargine, detemir, other insulinj |
| Chang, 2011^51^ | Case-Control Study | Taiwan | Patients with diabetes in Taiwan National Health insurance database from 2000 to 2007 | 66.13 | 63.25 | National cancer register | Non metformin users | All malignancy (1281/6385) | OR | No adjustment variables |
| Ferrara, 2011^52^ | Prospective cohort study | USA | Patients with diabetes in Kaiser Permanente Northern California (KPNC) Diabetes Registry from 1997 to 2005 | 64.3 | 53.4 | KPNC Cancer registry | Non metformin users | Prostate | AHR | Age, ever use of other diabetes medications, year of cohort entry, sex, race/ethnicity, income, current smoking, baseline HbA1c, diabetes duration, new diabetes diagnosis, creatinine, and congestive heart failure. |
|  |  |  |  |  |  |  |  | Breast |  |  |
|  |  |  |  |  |  |  |  | Lung |  |  |
|  |  |  |  |  |  |  |  | Colon |  |  |
|  |  |  |  |  |  |  |  | Uterus |  |  |
|  |  |  |  |  |  |  |  | Pancreas |  |  |
|  |  |  |  |  |  |  |  | Kidney/Pelvis |  |  |
|  |  |  |  |  |  |  |  | Colon |  |  |
|  |  |  |  |  |  |  |  | Malignant Melanoma |  |  |
| Lee, 2011^53^ | Retrospective cohort study | Taiwan | Patients with diabetes in Taiwanese National Health insurance database- median of Follow-up 3.8 yrs | 57.48 | 45.2 | A08-A14/ICD140-208 | Other hypoglicaemic drug users | All malignancy (339/15717) | AHR | Age group, gender, other oral medication Charlson comorbidity index scand duration of exposition to metformin |
|  |  |  |  |  |  | (151/A091) |  | Stomach (34/15412) |  |  |
|  |  |  |  |  |  | (153-154/093-A094) |  | Colon (56/15434) |  |  |
|  |  |  |  |  |  | (155/A095) |  | Liver (73/15451) |  |  |
|  |  |  |  |  |  | (157/A096) |  | Pancreas (28/15406) |  |  |
| Melbin, 2011^54^ | DIGAMI 2: Extension of randomized open label trial | Sweden | Patients with T2DM and suspected myocardial infarction. Median Follow-up 4.1yrs | 68.3 | 67 | Clinical documentation verified by an independent Adjudication Committee Events | Non metformin users | Cancer mortality (37/1073) | OR | No adjustment variables |
| Monami, 2011^55^ | Nested case-control study | Italy | Insulin treated type 2 diabetic patients in charge of University Diabetes Clinic-Florence- median of Follow-up 6.3 yrs | 68.2 | 51.7 | ICD9 codes (140-209) | Non metformin users | All malignancies (112/482) | AOR | CCS, glargine MDD, and total MDD of insulin |
| Nkontchou, 2011^56^ | Prospective cohort study. | France | Patient with T2DM in screening program for Hepatocellular carcinoma attending University Hospital referral center from 1998 to 2007- median follow-up 5.0 yrs | 61 | 53 | Histology or non invasive criteria | Non metformin users | Liver (39/100) | AHR | Age, platelet count, BMI, past alcohol abuse, diabetes duration |
| Yang, 2011^57^ | Prospective cohort study | Hong Kong-China | T2DM patients in Hong Kong Diabetes Registery from 1996-to 2005. - median of Follow-up 5.5 yrs | 55.43 | 49.2 | ICD9 codes (140-208) | Non metformin users | All malignancies (129/2658) | OR | No adjustment variables |
| Bo, 2012^58^ | Retrospective cohort study | Italy | Patients with T2DM attending Diabetic Clinic of S. Giovanni Battista Hospital- Turin form 1995 to 2000 | 67.4 | 52.2 | ICD9 codes (140-239) | Non hypoglicaemic drug users | Cancer mortality (49/2099) | AHR | For propensity score (Age, sex, diabetes duration, HbA1c, smoking, BMI, presence of: retinopathy, nephropathy, coronary or peripheral artery disease, other comorbidities, use of antihypertensive drugs and acetylsalicylic acid |
| Bodmer, 2012^59^ | Case-control study | UK | Patients with diabetes in General Practice Research Database (GPRD) from 1995 to 2009 | 70.2 | 63.3 | READ medical Codes (not listed) | Non metformin users | Colorectal (920/6439) | OR | No adjustment variables |
| Bodmer, 2012^60^ | Case-control study | UK | Patients with diabetes in General Practice Research Database (GPRD) from 1995 to 2009 | - | - | READ medical Codes (not listed) | Non metformin users | Pancreas (307/1654) | A OR | BMI, smoking, su, insulin, alcool consumption, diabetes duration |
| Lai, 2012^61^ | Retrospective cohort study | Taiwan | Patients with diabetes in Longitudinal Health Insurance Database from Taiwan National Health Insurance research database from 2000 to 2008 | 55.5 | 55.8 | Linking Longitudinal Health Insurance Database with register for catastrophic illness patients | Non metformin users | Liver (224/1939) | AHR | Sex, age, and comorbidities (including cirrhosis, alcoholic liver damage, hepatitis B and hepatitis C) |
| Ruiter, 2012^62^ | Retrospective cohort study | Netherland | Patients with hypoglycemic drug prescription-incident users) in PHARMO Record Linkage System from 1998 to 2008 | 63.25 | 47.1 | ICD9 codes (140-172; 174-209; 235-239) | Sulfonylureas users | All malignancies 1(/85289) | AHR | Age,sex,year of first prescription hospitalization before ogld unique drug |
|  |  |  |  |  |  | ICD9 codes (185) |  | Prostate (226/40131) |  |  |
|  |  |  |  |  |  | ICD9 codes (174-175) |  | Breast (424/45158) |  |  |
|  |  |  |  |  |  | ICD9 codes (157) |  | Pancreas (166/85289) |  |  |
|  |  |  |  |  |  | ICD9 codes (155) |  | Liver (31/85289) |  |  |
|  |  |  |  |  |  | ICD9 codes (153-154) |  | Colorectal (527/85289) |  |  |
|  |  |  |  |  |  | ICD9 codes (151) |  | Stomach (117/85289) |  |  |
|  |  |  |  |  |  | ICD9 codes (150) |  | Oesophagus (91/85289) |  |  |
|  |  |  |  |  |  | ICD9 codes (160-165) |  | Respiratory (454/85289) |  |  |
| Gregorio, 1999^63^ | Multicentre randomized, open-label controlled clinical trial | Italy | Patients with T2DM treated with a sub-massimal dose of Sulphonylureas | 75.6 | 47.1 | Reported as serious adverse events | No metformin | Brast cancer (1/92) | OR | No adjustment variables |
| Hanefield, 2004^64^ | Multicentre randomized, double-blind controlled clinical trial | Europe and North America | Patients with T2DM inadequately managed with Sulphonylureas alone. One years of follow-up. | 60.0 | 54.2 | Reported as serious adverse events | Pioglitazone | All malignancies (9/639) | OR | No adjustment variables |
| Schernthaner, 2004^65^ | Multicentre randomized, double-blind controlled clinical trial | Europe | Patients with T2DM inadequately managed with diet alone enrolled in 167 centers. One year of follow-up. | 56.5 | 55.2 | Reported as serious adverse events | Pioglitazone | All malignancies(9/1194) | OR | No adjustment variables |
| Goke, 2008^66^ | Extension of multicentre randomized, double-blind controlled clinical trial | Europe and America | Drug-naïve patients with T2DM. Two years of follow-up | 54 | - | Reported as serious adverse events | Vildagliptin | Cancer mortality (1/462) | OR | No adjustment variables |
| Lund, 2009^67^ | Randomized, double-blind controlled clinical trial | Denmark | Non obese patients with T2DM. One year of follow-up. | 63.3 | 61.4 | Reported as serious adverse events | Repaglinide | All malignancies(2/101) | OR | No adjustment variables |
| Aschener, 2010^68^ | Multicentre randomized, double-blind controlled clinical trial | Europe, America and Asia | Drug-naïve patients with T2DM. Six months of follow-up. | 56.0 | 46.3 | Reported as serious adverse events | Sitagliptin | Cancer mortality (1/894) | OR | No adjustment variables |
| Williams-Herman, 2010^69^ | Extension of multicentre randomized, double-blind controlled clinical trial | 18 countries worldwide | Patients with T2DM inadequately managed with diet and exercise enrolled in 117 centers. Two years of follow-up | 53.0 | - | Reported as serious advers events | Sitagliptin | All malignancies (18/543) | OR |  |
|  |  |  |  |  |  |  |  | Cancer mortality (1/543) | OR |  |
| Hense, 2011^70^ | Prospective cohort study | Germany | Patients with diabetes in German Statutory Health Insurance (SHI) system from 2003 to 2008. | 64.05 | 47.3 | Linking German Statutory Health Insurance system with Cancer Registry of the state of North-Rhine-Westphalia | No metformin only | All malignancies (1364/26742) | AHR | Sex, BMI, diabetes duration as a time-dependent variable, and medication at study entry. |
| Morden, 2011^71^ | Retrospective cohort study | USA | Patients with diabetes in Medicare database from 2003 to 2008. | 77.4 | 31.2 | ICD9 codes (140-239) | No metformin | All malignancies (5466/81681) | AHR | Age category, race/ethnicity, diabetes complications, obesity diagnosis, oral estrogen use, Part D lowincome subsidy (a poverty indicator), 14 Charlson comorbidities, and tobacco exposure diagnosis. |
|  |  |  |  |  |  | Clinical Classification Software Cancer Category (1740 1741 1742 1743 1744 1745 1746 1748 1749 1750 1759 2330) |  | Breast (553/56021) |  |  |
|  |  |  |  |  |  | Clinical Classification Software Cancer Category (1530 1531 1532 1533 1534 1535 1536 1537 1538 1539 1590 20910 20911 20912 20913 20914 20915 20916 2303) |  | Colon (428/81681) |  |  |
|  |  |  |  |  |  | Clinical Classification Software Cancer Category (185 2334) |  | Prostate (427/25660) |  |  |
| Chlebowsky, 2012^72^ | Prospective cohort study | USA | Postmenopausal women with T2DM. Mean of follow-up 11.8 yrs. | 64.0 | 0 | Medical reports and examinations validated according SEER criteria | Other hypoglicaemic drug users | Breast (807/3401) |  | Age, first-degree relative with breast cancer, benign breast disease, age at menarche, age at menopause, parity, age at first birth, education, No. of months of breastfeeding, smoking, alcohol consumption, body mass index, physical activity, duration of use of estrogen alone, duration of use of estrogen plus progesterone, bilateral oophorectomy, mammogram within 2 years of baseline, and stratified according to age (10-year age groups), hormone therapy trial randomization, dietary trial randomization or OS enrollment, enrollment onto Women’s Health Initiative extension, and race/ethnicity |
| Van Staa, 2012^73^ | Prospective cohort study | UK | Patients with diabetes in General Practice Research Database (GPRD) from 1997 to 2006 | 63.78 | 56.4 | Incident cancer in GPRD, HES and Cancer registry | Sulfonylureas users | All malignancies (1596/177737) | OR | No adjustment variables |
|  |  |  |  |  |  |  | Thiazolidinediones | All malignancies  (1018/141080) | OR | No adjustment variables |
|  |  |  |  |  |  |  | Insulin users | All malignancies (1145/132713) | OR | No adjustment variables |
