# Supplementary material for: Metformin Therapy and Risk of Cancer in Patients with Type 2 Diabetes: Systematic Review
Source: PLoS One. 2013 Aug 2;8(8):e71583. doi: 10.1371/journal.pone.0071583 (PMC3732236; doi:10.1371/journal.pone.0071583)
Supplement: Table S3 — (DOC) [file pone.0071583.s009.doc]

**Table S2: Results of additional meta-analysis**

| **Cancer site** |  | **Observational studies** |  |  | **RCTs** |  |
| --- | --- | --- | --- | --- | --- | --- |
|  | **Studies (n°)** | **Patients (n°)** | **OR (95%CI)** | **Patients (n°)** | **Studies (n°)** | **OR (95%CI)** |
| Lung cancer | 4 | 505466 | 0.83 (0.36-0.81) | 2 | 6576 | 0.73(0.37-1.45) |
| Ovary cancer | 1 | 565 | 0.69(0.43-1.10) | 2 | 2965 | 0.32(0.06-1.66) |
| Kidney/pelvis cancer | 1 | 252467 | 1.30(1.00-1.60) | 2 | 6576 | 0.38(0.10-1.46) |
| Melanoma cancer | 1 | 252467 | 0.80(0.60-1.10) | 2 | 6576 | 0.75(0.21-2.66) |
| Uterus cancer | 1 | 117603 | 0.90(0.80-1.20) | 2 | 2956 | 0.87(0.36-2.14) |
| Bladder cancer | 1 | 191223 | 0.99(0.70-1.39) | 2 | 6576 | 1.08(0.45-2.59) |
